# Supplementary material for: Antimicrobial and Mechanical Properties of Ag@Ti3C2Tx-Modified PVA Composite Hydrogels Enhanced with Quaternary Ammonium Chitosan
Source: Polymers (Basel). 2023 May 18;15(10):2352. doi: 10.3390/polym15102352 (PMC10224062; doi:10.3390/polym15102352)
Supplement: Supplementary file 1 [file polymers-15-02352-s001.zip › polymers-2381157-supplementary.pdf]

Supplementary Materials

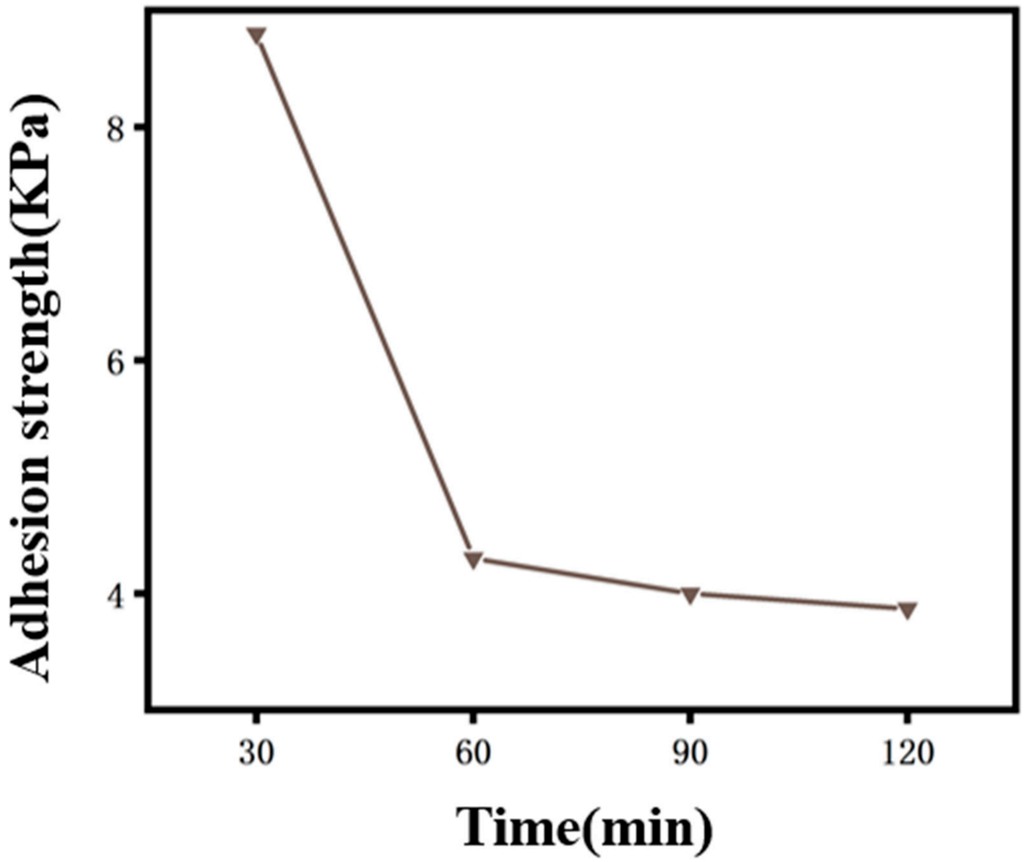

Figure S1. Time-adhesion strength curves of Ag@M-H-PVA hydrogels (Ag@M3wt%, HACC9wt%) under wet conditions.
